# Seven-day triple therapy is sufficient to eradicate infection caused by *Helicobacter pylori* without *23S rRNA* point mutation

**DOI:** 10.1097/MD.0000000000026133

**Published:** 2021-05-28

**Authors:** Jung Wook Lee, Su Jin Kim, Cheol Woong Choi, Hyeong Jin Kim, Dae Hwan Kang, Hyung Wook Kim, Su Bum Park, Hyeong Seok Nam, Dae Gon Ryu

**Affiliations:** Department of Internal Medicine, Pusan National University School of Medicine and Research Institute for Convergence of Biomedical Science and Technology, Pusan National University Yangsan Hospital, Yangsan, Republic of Korea.

**Keywords:** clarithromycin, *Helicobacter pylori*, polymerase chain reaction, triple therapy

## Abstract

Tailored therapy based on dual priming oligonucleotide-based polymerase chain reaction (DPO-PCR) can be considered an alternative to overcome the low eradication rate in high clarithromycin-resistance areas. The triple therapy (TT) duration of the tailored approach in most studies was 7 days for patients without point mutation. However, recent western guidelines have recommended a treatment duration of 14 days. The aim of this study was to compare the success rate of 7 and 14 days of TT for eradicating *Helicobacter pylori* without point mutation, as determined by DPO-PCR.

Between Feb 2016 and Feb 2019, medical records of patients who underwent DPO-PCR were reviewed. Patients without point mutation as determined by DPO-PCR were enrolled in this study. The eradication success rate and adverse events were evaluated.

A total of 366 patients without A2142G and A2143G point mutation were enrolled. The success rates of 7-day and 14-day TT were 88.4% (168/190) and 85.9% (151/176) by intention to treat analysis (*P* = .453) and 90.8% (168/185) and 90.4% (151/167) by per-protocol analysis (*P* = .900), respectively. The adverse event rates showed no significant difference between the 2 groups.

In patients without point mutation based on DPO-PCR results, 7-day TT is as effective as 14-day TT. Therefore, 7 days may be considered as a cost-effective treatment duration in Korea.

## 1 Introduction

*Helicobacter pylori* (*H pylori*) causes gastric cancer, mucosa-associated lymphoid tissue (MALT) lymphoma, peptic ulcers, and chronic atrophic gastritis.^[1]^ The International Agency for Research on Cancer of the World Health Organization categorized *H pylori* as a Group 1 carcinogen in 1994 and reconfirmed this categorization in 2009.^[2,3]^ Therefore, all patients who underwent endoscopic resection for early gastric cancer are recommended to be tested and treated for *H pylori* infection. Successful eradication can also lead to curative response in the majority of patients with gastric MALT lymphoma and reduce the recurrence rate of peptic ulcer.^[4,5]^

Triple therapy (TT) composed of a proton pump inhibitor (PPI), amoxicillin, and clarithromycin is considered as the first-line eradication regimen in Korea and Japan.^[6,7]^ However, the efficacy of TT has gradually decreased in Korea because of antibiotic-resistant *H pylori* strains.^[8,9]^ Especially, the success rate of TT is significantly different according to clarithromycin resistance.^[10]^ Therefore, western guidelines have recommended restricting the use of TT to known areas with low clarithromycin resistance (approximately <15–25%).^[11–13]^ A regimen based on the antimicrobial susceptibility test is an ideal approach to increase the success rate of *H pylori* eradication. However, *H pylori* culture and antimicrobial susceptibility tests are time-consuming, expensive, and often difficult to perform; also, the success rate of culture test is low.^[14]^ DPO-PCR can conveniently and rapidly detect the mutation of *23S ribosomal ribonucleic acid* (*rRNA*), which results in clarithromycin resistance.^[15]^ Tailored therapy based on DPO-PCR can guide a clinician to decide the first-line regimen individually for a patient belonging to an area with high clarithromycin resistance. TT can be eventually used as the first-line regimen for *H pylori* treatment without A2142G and A2143G point mutations.

However, the duration of TT is still debatable. The recommended duration varies according to the guidelines available in different countries. For example, 7 days of treatment is recommended in Japan while 7 to 14 days are recommended in Korea.^[6,7]^ On the other hand, recent guidelines have recommended a treatment duration of 14 days.^[11–13,16]^ In the present study, we aimed to determine the optimal duration of TT for *H pylori* eradication without introducing point mutation in *23S rRNA* by comparing the eradication rates between 7- and 14-day treatment duration of TT.

## 2 Methods

The medical records of patients who received DPO-PCR (Seeplex *H pylori*-ClaR ACE Detection; Seegene Inc., Seoul, Korea) during endoscopy at the Pusan National University Yangsan Hospital, Republic of Korea between January 2016 and February 2018 were retrospectively reviewed. During the study period, *H pylori* infection was confirmed by performing DPO-PCR in 487 patients. DPO-PCR can detect A2142G and A2143G point mutation in the *23S rRNA* gene. The exclusion criteria were as follows: younger than 18 years of age, *H pylori* infection with A2142G and/or 2143G mutation, patients who have undergone previous *H pylori* eradication treatment, history of gastrectomy, severe concurrent disease, history of allergic reaction to antibiotics, and history of antibiotic use within 4 weeks of study commencement. After exclusion, 366 patients were enrolled. The present study was approved by the ethics committee of the hospital where this study was performed.

### 2.1 Diagnosis of *H pylori* infection

Gastric biopsy specimens were obtained from the antrum and body of the patients by endoscopy. DPO-PCR was performed to diagnose *H pylori* infection by detecting the deoxyribonucleic acid (DNA) extracted from gastric biopsy specimens. A2142G and/or A2143G mutation associated with clarithromycin resistance were identified by polymerase chain reaction (PCR) amplification of a portion of the *23S rRNA* gene.

The success of *H pylori* eradication was assessed by the ^13^C-urea breath test (UBiT-IR300; Otsuka Electronics Co. Ltd., Tokyo, Japan) at least 4 weeks after completing the eradication process. All the patients were requested to swallow a pill containing 100 mg ^13^C-urea (UBiTkit; Otsuka Pharmaceutical Co. Ltd., Tokyo, Japan) with 100 mL water after at least 4 hours of fasting. Two breath samples were collected before and 20 minutes after the ingestion of the ^13^C-urea pill. A value of ^13^CO_2_ >2.5‰ was considered positive for confirming *H pylori* infection.^[17]^

### 2.2 Eradication regimen

The patients were treated with TT that consisted of a proton pump inhibitor (rabeprazole 20 mg or esomeprazole 20 mg), amoxicillin 1 g, and clarithromycin 500 mg twice daily for 7 or 14 days. At least 4 weeks from treatment completion, the patients visited the hospital for undergoing the urea breath test that assessed whether the TT was successful or not. Compliance and adverse events related with the drug were evaluated by a physician. Consumption of <80% of the prescribed medicine based on the pill count specified in the medical records was defined as noncompliance.

All the patients in whom *H pylori* eradication failed after the completion of TT were recommended to receive bismuth quadruple therapy (BQT) that consisted of a proton pump inhibitor (rabeprazole 20 mg or esomeprazole 20 mg) twice daily, metronidazole 500 mg 3 times daily, tetracycline 500 mg, and 300 mg potassium bismuth citrate 4 times daily for 14 days as the second-line *H pylori* eradication therapy. The eradication success rate of the BQT was also determined using the ^13^C-urea breath test at least 4 weeks after treatment.

### 2.3 Statistical analysis

The eradication and adverse event rates were compared between 7 day- and 14 day-treatment groups. Eradication rate analysis was performed on the basis of intention-to-treat (ITT) and per-protocol (PP). ITT analysis included the data of all the patients enrolled in this study, while PP analysis included the data of only those patients who consumed ≥80% of the prescribed medications. Categorical variables were analyzed using the chi-square test or Fisher exact test and continuous variables were analyzed using the Student *t* test. A *P*-value <.05 was considered statistically significant. Statistical calculations were performed using the Statistical Package for the Social Sciences version 21.0 for Windows (IBM Corp., Armonk, NY).

## 3 Results

**Figure 1:**
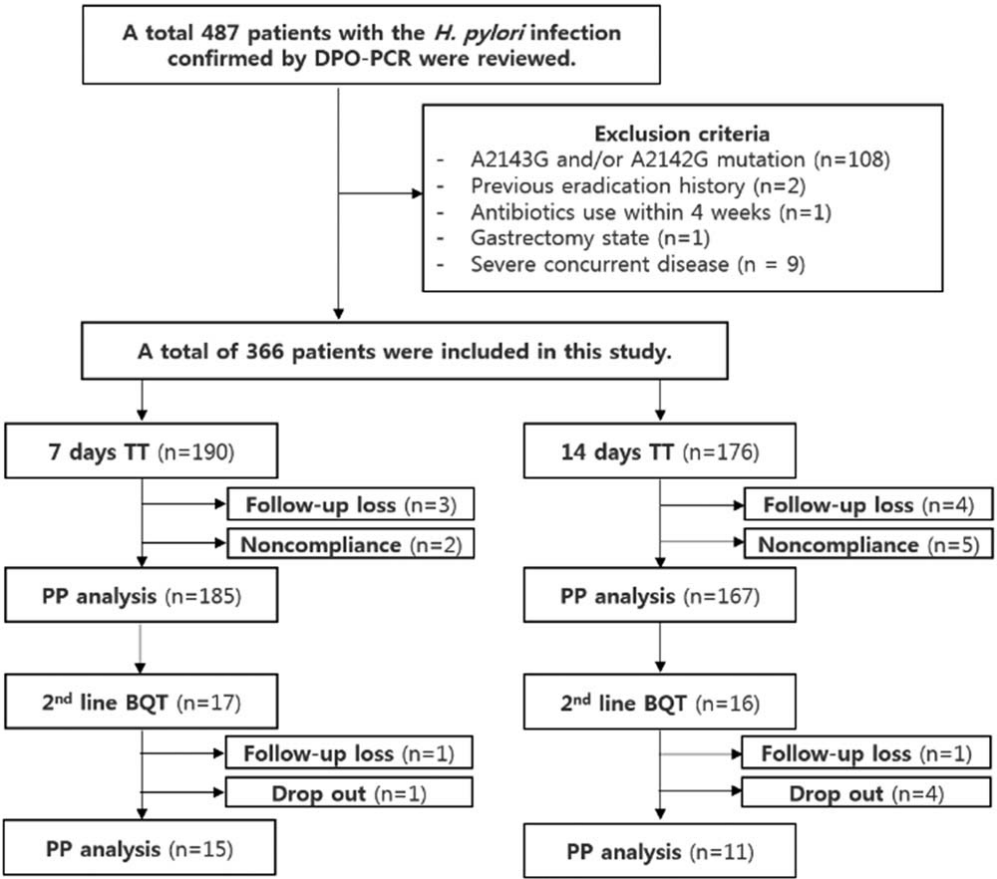
Flowchart of patients. BQT = bismuth based quadruple therapy, DPO-PCR = dual priming oligonucleotide-based polymerase chain reaction, PP = per-protocol, TT = triple therapy.

During the study period, *H pylori* infection was confirmed in 487 consecutive patients by DPO-PCR. The clarithromycin-resistant strain of *H pylori* was found in 108 patients (22.2%). Among 379 patients infected with *H pylori* without A2142G and/or A2143G point mutation, 13 patients were excluded because of a previous history of *H pylori* eradication treatment (n = 2), history of gastrectomy (n = 1), severe concurrent disease (n = 9), and history of antibiotic use within 4 weeks of current treatment (n = 1). Finally, 366 patients were enrolled in this study, of which, 190 received TT for 7 days and 176 received TT for 14 days, respectively. Three patients in the 7-day TT group and 4 patients in the 14-day group were lost to follow-up after eradication (Fig. 1). There was no significant difference in the clinical characteristics between the 2 groups (Table 1).

**Table 1:** Baseline characteristics of patients.

	7 days group (n = 190)	14 days group (n = 176)	*P* value
Mean age, y (±SD)	58.3 ± 12.6	58.2 ± 10.9	.963
Sex			.063
Male, n (%)	146 (76.8)	120 (68.2)	
Female, n (%)	44 (23.2)	56 (31.8)	
Smoking, n (%)	58 (30.5)	48 (27.3)	.493
Alcohol, n (%)	85 (42.5)	71 (41.8)	.886
Cause of treatment, n (%)			.219
Peptic ulcer	82 (43.2)	57 (32.4)	
ER for EGC or adenoma	86 (45.3)	93 (52.8)	
MALT lymphoma	6 (3.2)	11 (6.3)	
Gastric polyp	1 (0.5)	1 (0.6)	
Atrophic gastritis	15 (7.9)	14 (8.0)	

### 3.1 Eradication rates of *H pylori* and adverse events

The eradication rates of *H pylori* based on the ITT analysis showed no significant difference in terms of the duration of TT 88.4% (168/190) in the 7-day TT group and 85.9% (151/176) 14-day TT group (*P* = .453). The PP analysis results revealed that there was no significant difference in the eradication rates between the 2 groups (90.8% [168/185] and 90.4% [151/167], *P* = .900) (Table 2).

**Table 2:** Eradication rates in each groups.

	7 days group	14 days group	*P* value
1st line TT eradication success, n/total (%)			
ITT	168/190 (88.4)	151/176 (85.9)	.453
PP	168/185 (90.8)	151/167 (90.4)	.900
2nd line BQT eradication success, n/total (%)			
ITT	13/17 (76.5)	9/16 (56.3)	.218
PP	13/15 (86.7)	9/11 (81.8)	1.000
Overall success rate, n/total (%)	181/190 (95.3)	160/176 (90.9)	.099

Adverse events including bitter taste, nausea or vomiting, diarrhea, and abdominal discomfort or pain, and urticaria were reported in 15.5% (29/187) in the 7-day TT group and 18.6% (32/172) in the 14-day TT group (Table 3). Of 61 patients, 2 patients in the 7-day TT group and 5 patients in the 14-day TT group showed noncompliance by failing to ingest >80% of the prescribed medication as a result of the adverse events. Three patients (1 in the 7-day TT group and 2 in the 14-day TT group) stopped taking medication because of urticaria and pruritus. Four patients (1 in the 7-day TT group and 3 in the 14-day TT group) stopped the treatment due to severe gastrointestinal symptoms such as vomiting, persistent diarrhea, and abdominal pain. The symptoms of all the patients who experienced adverse events improved after the treatment was stopped and after taking symptom-control medications without hospitalization.

**Table 3:** Adverse events associated with the triple therapy.

	7 days group (n = 187)	14 days group (n = 172)	*P* value
Bitter taste, n (%)	16 (8.6)	17 (9.9)	.664
Nausea/Vomitting, n (%)	1 (0.5)	2 (1.2)	.514
Diarrhea, n (%)	3 (1.6)	3 (1.7)	.918
Abdominal discomfort or pain, n (%)	8 (4.3)	8 (4.7)	.864
Urticaria, n (%)	1 (0.5)	2 (1.2)	.514
Overall, n/total (%)	29/187 (15.5)	32/172 (18.6)	.435

The second-line BQT was recommend for 33 patients in whom eradication of *H pylori* infection failed after completing TT. Of the 33 patients, 5 patients (1 in the 7-day TT group and 4 in the 14-day TT group) refused to receive the BQT. Therefore, 26 patients received second-line BQT. Two patients (1 in each group) were lost to follow-up. For PP analysis, the eradication rates of BQT were 86.7% (13/15) in the 7-day TT group and 81.8% (9/11) in the 14-day TT group (*P* = 1.000). Finally, the overall *H pylori* eradication rates were not significantly different (95.3% [181/190] in the 7-day TT group and 90.9% [161/176] in the 14-day TT group [*P* = .099]) (Table 2).

## 4 Discussion

In our study, the eradication rates of *H pylori* infection based on ITT were 88.4% and 85.9%, and based on PP analysis were 90.8% and 90.4%, respectively, in the 7-day and 14-day treatment groups. The incidence of adverse events was not significantly different. There was no serious adverse event associated with the medication. A few Korean studies have evaluated the eradication rate after 7 or 14 days of TT in patients with *H pylori* infection without *23S RNA* point mutation; the eradication rates reported were 95% to 98% for the 7-day treatment duration and 92% for the 14-day treatment duration.^[18–20]^ Both the treatment durations in the present study also reached the appropriate therapeutic treatment duration based on ITT and PP analyses, which was similar to that reported by previous Korean studies.^[21]^ Although western guidelines recommend a 14-day treatment duration to overcome antibiotic resistance, several meta-analysis studies have demonstrated the effectiveness of a 7-day tailored therapy for eradicating *H pylori* infection.^[12,13,22]^

*H pylori* is mostly associated with gastric cancer, MALT lymphoma, and peptic ulcer.^[23]^*H pylori* infection is diagnosed in more than half of the world's population and a nationwide study performed in Korea also reported that the *H pylori* seropositivity rate was 51.0%.^[24]^ The treatment of *H pylori* infection showed therapeutic or preventive benefits regarding the associated disease. Especially, the concern has arisen recently regarding the preventive effect about gastric cancer.^[25,26]^ The standard TT regimen includes a PPI, clarithromycin, and amoxicillin as the current empirical first-line therapy for *H pylori* treatment in Korea. The efficacy of the first-line 7-day TT has been declining to <80% because of the formation of an increasing number of clarithromycin-resistant strains. In Korea, antibiotic resistance rates of *H pylori* have increased for amoxicillin (6.3–14.9%) and clarithromycin (17.2–23.7%) from 2003 to 2012.^[9]^ Most western guidelines recommend other eradication regimens such as bismuth quadruple or concomitant therapy in an area with high (>15%) clarithromycin resistance.^[11–13]^ A recent large-scale, nationwide, randomized controlled trial assessing the efficacies of a 10-day concomitant therapy (CT) and 10-day sequential therapy (ST) versus a 7-day TT reported that the 10-day CT and 10-day ST were superior to the 7-day TT as the empirical first-line *H pylori* treatment in Korea.^[27]^ Therefore, 10-day CT and 10-day ST can be included as empirical regimens in new Korean guidelines for the treatment of *H pylori* infection.

Alternatively, clarithromycin susceptibility test is recommended to overcome the failure of empirical therapy in an area with high clarithromycin resistance.^[13]^ Although the antibiotic resistance test by culture can result in an ideal outcome, this test is not only difficult but also non-available in most centers. As an alternative, molecular methods such as DPO-PCR can identify key mutations known to be responsible for antibiotic resistance.^[14]^ Antibiotic susceptibility-guided *H pylori* eradication is defined as tailored therapy. Compared to CT and ST, a tailored therapy has several benefits. First, a tailored therapy can help in avoiding antibiotic overuse in CT and ST by selecting a patient infected with a clarithromycin-resistant *H pylori* strain, which can cause eradication failure of CT and ST.^[20]^ Second, repeating the same antibiotics included in the previous treatment can decrease the eradication rate.^[13]^ However, the bismuth quadruple, which is the most preferred rescue regimen after the failure of CT and ST, includes other drugs and metronidazole that are also included in CT and ST. Therefore, a recent meta-analysis reported that tailored therapy showed better outcome for *H pylori* eradication compared with an empirical treatment.^[22]^

A 14-day treatment duration compared with 7 days can be helpful for eradicating *H pylori* with antibiotic resistance. However, *H pylori* without *23S RNA* point mutation has a low possibility of clarithromycin resistance. Therefore, the benefit of a longer duration treatment is limited in these situations. A 14-day TT may result in the overuse of antibiotics for patients with *H pylori* infection without *23S RNA* point mutation. In addition, the BQT is the preferred regimen as a rescue treatment after the failure of TT and an optimum duration of this regimen for *H pylori* eradication is 14 days. The failure of 14-day TT can decrease the willingness to receive a 14-day rescue regimen compared with 7-day TT.

Our study had several limitations. First, it was a retrospective study. Patients received different PPIs, which could have affected the eradication rate. The meta-analysis reported that no difference in the eradication rate was observed among new-generation PPIs, although new-generation PPIs showed a better eradication rate than the old-generation ones.^[28]^ A rabeprazole and esomeprazole that were used as TT in this study are new-generation PPIs and showed no difference in the eradication rates of TT according to the PPIs (90.0% in rabeprazole user and 91.6% in esomeprazole user, *P* = .601). Second, a retrospective, single-center study might have caused selection bias. The patients of our hospital may not be representative of all Korean patients. There is region-based antimicrobial susceptibility difference in Korea. Therefore, a prospective, multicenter study is needed to confirm this conclusion. Third, an antimicrobial susceptibility test based on culture was not performed. Amoxicillin and metronidazole resistance data can be helpful to further understand the results of this study.

In summary, although recent guidelines recommend a duration of 14 days for TT consisted of PPI (standard dose), amoxicillin (1 g), and clarithromycin (500 mg) twice daily, a 7-day TT is as effective as 14-day therapy in patients without *23S RNA* mutation based on the DPO-PCR detection method. For a tailored eradication therapy, 7 days may be considered a cost-effective treatment duration in Korean patients infected by *H pylori* with very low possibility of clarithromycin resistance.

## Author contributions

**Conceptualization:** Jung Wook Lee, Su Jin Kim, Cheol Woong Choi, Dae Hwan Kang, Hyung Wook Kim, Su Bum Park, Hyeong Seok Nam, Dae Gon Ryu.

**Data curation:** Jung Wook Lee, Su Jin Kim, Hyeong Jin Kim, Hyung Wook Kim, Su Bum Park, Hyeong Seok Nam, Dae Gon Ryu.

**Formal analysis:** Jung Wook Lee, Su Jin Kim, Hyeong Jin Kim.

**Funding acquisition:** Jung Wook Lee, Su Jin Kim.

**Investigation:** Jung Wook Lee, Su Jin Kim.

**Methodology:** Jung Wook Lee, Su Jin Kim.

**Project administration:** Jung Wook Lee, Su Jin Kim.

**Resources:** Jung Wook Lee, Su Jin Kim.

**Software:** Jung Wook Lee, Su Jin Kim.

**Supervision:** Jung Wook Lee, Su Jin Kim.

**Validation:** Jung Wook Lee, Su Jin Kim.

**Visualization:** Jung Wook Lee, Su Jin Kim.

**Writing – original draft:** Jung Wook Lee, Su Jin Kim.

**Writing – review & editing:** Jung Wook Lee, Su Jin Kim, Cheol Woong Choi, Dae Hwan Kang.
